# Supplementary material for: The Transcriptional Regulation of Genes Involved in the Immune Innate Response of Keratinocytes Co-Cultured with Trichophyton rubrum Reveals Important Roles of Cytokine GM-CSF
Source: J Fungi (Basel). 2022 Oct 31;8(11):1151. doi: 10.3390/jof8111151 (PMC9693189; doi:10.3390/jof8111151)
Supplement: Supplementary file 1 [file jof-08-01151-s001.zip › jof-1982625-supplementary.pdf]

Table S1. Oligonucleotide sequences used for RT-qPCR analysis.

Gene ID	Gene Product Name	Oligonucleotide sequence	Amplicon	Reference
**CSF2**	Colony Stimulating Factor 2	F:5′-TCTGCTTGTCATCCCCTTTG-3′<br>R:5′-ATGACCATCCTGAGTTTCTAGC-3′	122 pb	[1]
**RNASE7**	Ribonuclease A Family Member 7	F:5′- GGAGTCACAGCACGAAGACCA-3′<br>R:5′- CATGGCTGAGTTGCATGCTTGA-3′	235 pb	[2]
**SLC11A1**	Natural resistance-associated macrophage protein 1	F:5′- CATCTCCGTCTCCCAAAGTG-3′<br>R:5′- TGGATGCTTTAGAGACTTCGTG-3′	144 pb	[3]
**KRT1**	Keratin 1	F:5′- CCTTACAGCACTCTACCAAGC-3′<br>R:5′-TCTGGGCTATATCCTCGTACTG-3′	149 pb	[3]
**FLG**	Filaggrin	F:5′- AGCAATCGGTAAATAGGTCTGG-3′<br>R:5′- CCTCATTTCGTGTTTGTCTGC-3′	126 pb	[3]
**TLR4**	Toll Like Receptor 4	F: 5′-TCTACAAAATCCCCGACAACC-3′<br>R: 5′- TGTCTGGATTTCACACCTGG-3'	143 pb	This paper
**GAPDH**	Glyceraldehyde-3-phosphate dehydrogenase	F: 5′-AATCCCATCACCATCTTCCAG-3′<br>R: 5′-GAGCCCCAGCCTTCTCCAT-3′	118 pb	[4]
**ACTB**	Beta-actin	F: 5′-GTTGCGTTACACCCTTTCTTG-3′<br>R: 5′-TGCTGTCACCTTCACCGTTC-3′	154 pb	[5]
